# A randomized controlled trial undertaken to test a nurse-led weight management and exercise intervention designed for people with serious mental illness who take second generation antipsychotics

**DOI:** 10.1111/jan.12012

**Published:** 2012-09-14

**Authors:** Kim Usher, Tanya Park, Kim Foster, Petra Buettner

**Affiliations:** Associate Dean of Graduate Research Studies, School of Nursing, Midwifery & Nutrition, James Cook UniversityCairns, Queensland, Australia; School of Nursing, Midwifery & Nutrition, James Cook UniversityCairns, Queensland, Australia; Mental Health Nursing Sydney Nursing School, University of SydneySydney, New South Wales, Australia; School of Public Health, Tropical Medicine and Rehabilitation Sciences, James Cook UniversityTownsville, Queensland, Australia

**Keywords:** metabolic syndrome, nursing, second generation antipsychotic medications, serious mental illness, weight gain

## Abstract

**Aim:**

To test the effect of a nurse-led intervention on weight gain in people with serious mental illness prescribed and taking second generation antipsychotic medication.

**Background:**

Weight gain and obesity has reached epidemic proportions in the general population with the prevalence of Metabolic Syndrome reaching 20–25% of the global population. People with serious mental illness are at even higher risk, particularly those taking second generation antipsychotic medication.

**Design:**

An experimental randomized controlled trial was undertaken.

**Method:**

The control group received a 12-week healthy lifestyle booklet. In addition to the booklet, the intervention group received weekly nutrition and exercise education, exercise sessions, and nurse support. Participants (*n* = 101) were assessed at baseline and 12 weeks. Data were collected between March 2008–December 2010. Seven outcome measures were used: body measurements included girth (cm), weight (kg), height (cm), and body mass index (kg/m^2^); questionnaires included the medication compliance questionnaire, the Drug Attitude Inventory, the Liverpool University Neuroleptic Side Effect Rating Scale, and the Medical Outcomes Study Short Form 36. Differences in primary outcome measures between baseline and 12 weeks follow-up were compared between intervention and control groups using standard bi-variate statistical tests. The study was conducted between 2008–2010.

**Results:**

The analysis of outcome measures for the control group (*n* = 50) and intervention group (*n* = 51) was not statistically significant. There was a mean weight change of −0·74 kg at 12 weeks for the intervention group (*n* = 51), while the control group (*n* = 50) had a mean weight change of −0·17 kg at 12 weeks.

**Conclusion:**

The results were not statistically significant.

## Introduction

While antipsychotic medication remains the mainstay of treatment for schizophrenia and other serious mental illnesses, the increased use of the newer second generation antipsychotics has led to concerns about their association with weight gain and other metabolic symptoms (Usher *et al*. 2009). The second generation antipsychotics are known to be linked to marked and predictable weight gain which is rapid in the initial period (usually the first 4–12 weeks) after commencement of the medication followed by a period where it continues but at a reduced rate. A meta-analysis of weight change by Allison *et al*. (1999) identified the following weight gain over 10 weeks of treatment with a standard dose of the following second generation antipsychotic medication: Clozapine, 4·45 kg; Olanzapine, 4·15 kg; Risperidone, 2·10 kg; and Ziprasidone, 0·04 kg. After the initial period the weight gain continues at a lower level over a prolonged period of time (Tschoner *et al*. 2007). The weight gain linked to second generation antipsychotic drugs is typically associated with abdominal obesity (McEvoy *et al*. 2005), and enhanced adiposity, which is linked to increased morbidity and mortality and reduced quality of life (McEvoy *et al*. 2005). These changes, along with hypertension, hyperlipidaemia, hyperglycaemia, and abdominal obesity, make up what is referred to as metabolic syndrome. Metabolic syndrome is known to lead to an increased risk of diabetes and cardiovascular disease (IDF 2006). Australian studies report prevalence rates of metabolic syndrome for people with serious mental illness ranging between 51–68% (Tirupati & Chua 2007, Brunero *et al*. 2009, John *et al*. 2009). Thus, weight gain and metabolic disturbance linked to the second generation antipsychotic medication has become a major concern for clinicians and consumers (Wand & Murray 2008). Interventions have been introduced in an attempt to manage the weight gain linked to these medications, including lifestyle, education, weight loss medications, and exercise have all been tried and evaluated. Research to date indicates significantly greater weight reduction in lifestyle intervention groups vs. pharmacological intervention groups or standard care groups (Littrell *et al*. 2003, Vreeland *et al*. 2003, Weber & Wyne 2006).

## Background

Early intervention for weight gain with second generation antipsychotic medication should be the first priority when a person with a serious mental illness commences taking SGA medications and when antipsychotic medication changes occur. Interventions at that time should include healthy lifestyle education, reduced calorie intake, and increased exercise (Citrome *et al*. 2005, Tschoner *et al*. 2007). However, for those who have been taking second generation antipsychotic medication for some time and have put on a significant amount of weight as a result, several interventions have been implemented to help with weight management and reduction.

Single mode intervention studies range from 12–36 weeks duration and tend to be drug-based. For example, the administration of metformin has been successful in arresting weight gain and leading to significant BMI reductions in many participants (Morrison *et al*. 2002). Education programmes that deliver information on nutrition, wellness, fitness, and exercise, have also had some success. A 12-week study by Ball *et al*. (2001), included 22 participants with serious mental illness of whom 11 attended weekly Weight Watcher group meetings, three attended weekly exercise sessions, while a comparison group (*n* = 11) did not participate in the groups but maintained their medication. The study reported successful outcomes with weight loss ranging from 0–8·2 kg. Other studies have found disparate results. For example, Lindenmayer *et al*. (2009) found a significant mean weight and BMI reduction of approximately 2 kg over 9 months but a similar study conducted over 6 months reported no weight loss.

Multimodal interventions range from 3–18 months in duration and used a combination of approaches such as education, exercise, dietary management, and lifestyle programmes (McCloughen & Foster 2011). For example, 12-week programmes with educational and behavioural components have achieved significant weight reductions (Melamed *et al*. 2008, Kwon *et al*. 2006, Skouroliakou *et al*. 2009). Studies using amantadine (a dopamine agaonist that decreases appetite) and metformin combined with lifestyle education, diet and exercise, appeared to achieve weight stabilization in the intervention group rather than weight loss and weight gain in the control group (Graham *et al*. 2005). However, weight loss was achieved in a study with a larger number of participants (Wu *et al*. 2008). Studies conducted over longer duration tended to have better outcomes. For example, the study by Poulin *et al*. (2007), conducted over 18 months with education followed by structured weekly fitness classes, had a significant weight and BMI reduction.

In summary, significantly greater weight reduction has been found in lifestyle intervention groups vs. pharmacological intervention groups or standard care groups since the landmark DPP study in 1996–1998 (Ball *et al*. 2001, Littrell *et al*. 2003, Vreeland *et al*. 2003, Evans *et al*. 2005, Knowler *et al*. 2005, Weber & Wyne 2006). However, the outcomes so far are limited by factors such as the small number of studies, small sample sizes, short study duration, and by variability in the interventions, including their intensity and duration. Additional research that distinguishes between weight gain prevention and weight gain reversal has been recommended (Faulkner *et al*. 2007).

## The study

### Aim

The aim of this study was to test the effect of a nurse-led intervention on weight gain in people with serious mental illness (SMI) prescribed and taking second generation antipsychotic medication.

### Design

An experimental randomized controlled trial was undertaken.

### Participants

There were 104 people recruited to participate in the study. Two people attended the information session only and one person left the study due to medical reasons, resulting in a total of 101 participants; 51 in the control group and 50 in the intervention group. Inclusion criteria for the study was developed and implemented as a measure of control. Inclusion criteria were: a diagnosis of serious mental illness, 18 years of age or older, not currently psychotic, prescribed and taking second generation antipsychotic medication, living in North Queensland, and able to speak and read English. For the purpose of the study the term ‘serious mental illness’ was used to collectively describe the illnesses for which Second generation antipsychotic medication are usually prescribed. These include schizophrenia, bipolar disorder, and other psychotic disorders. Participants were recruited to the study via posters displayed at local community mental health services, non-government organizations (NGOs), word of mouth and as a result of presentations on the topic in the local area. Participants were from a combination of five local mental health services including NGOs. All participants had a diagnosis of a serious mental illness, were over 18 years of age, were assessed by the team as well and thus able to consent to participate, and able to read English.

### Data collection

Data were collected by the researchers between March 2008–December 2010. Demographic data collected from participants included: age, gender, ethnicity, diagnosis, current medication. Outcome measurements collected included weight, height and girth measurements, and BMI. All participants had baseline outcome measures assessed by a research assistant which were repeated at 12 weeks. The participants in the control group had identical measures taken at the same time periods as the intervention group.

### Instruments

The following survey instruments were used to collect outcome data at baseline and 12 weeks in both groups. The survey instruments were chosen to help better understand the associated effects a nurse-led healthy lifestyle intervention on other quality of life factors such as general health and well-being and medication side effects. Adherence to medication was also measured to indicate the extent to which the cohort actually followed the recommended medication regime.

The Medication Compliance Questionnaire is a measure of medication compliance with a 7-point scale ranging from ‘complete refusal’ to ‘active participation’ used to assess medication compliance in the participants (Kemp *et al*., 1996). The medication compliance questionnaire is scored according to the response given: complete refusal = 1; partial refusal = 2; reluctant acceptance = 3; occasional reluctance about treatment = 4; passive acceptance = 5; moderate participation = 6; active participation = 7.

The Drug Attitude Inquiry-10 (DAI-10) is a self-report inventory on the subjective effects of neuroleptic medications. The person is asked to answer 10 questions choosing either ‘true’ or ‘false’. The DAI-10 is reported as having good internal consistency with high test–retest reliability (Awad 1993). The DAI-10 questionnaire is scored according to the true or false answer given, as either +1 or −1. The final score is the sum of the total of pluses and minuses. A positive total score means a positive subjective response (compliant). A negative score means a negative subjective response (non-compliant).

The Liverpool University Neuroleptic Side Effect Rating Scale is a self-report questionnaire for the reporting of general neuroleptic side effects, with 51 questions which can be grouped under the following categories: extrapyramidal side effects, psychic side effects, hormonal side effects, anticholinergic side effects, miscellaneous side effects, other autonomic side effects, allergic reactions, and red herrings. Using a 5-point Likert scale, the person rates each item as ‘not at all, very little, a little, quite a lot or very much’. The Liverpool University Neuroleptic Side Effect Rating Scale has been used as a useful rating scale to assess subjective tolerability of antipsychotics in previous studies (Kim *et al*. 2006). To obtain a final score each category is added together (excluding the red herring score) and then the red herring score is subtracted for the final score to be obtained. The final score is then classified: 0–40 = low; 41–80 = medium; 81–100 = high; >101 = very high. A red herring score above 20 is considered high and may indicate individuals who over score in general.

The Medical Outcomes Study Short Form 36 (SF-36) is a generic well-validated multipurpose health measure (Turner-Bowker *et al*. 2002). The questionnaire measures indicators of health including: behavioural function and dysfunction, distress and well-being, objective and subjective ratings, and favourable and unfavourable self-evaluations of general health (Ware *et al*. 2002). Validity and reliability of the tool is well-documented and the SF-36 is considered a useful benchmark when comparing well and unwell populations to estimate the burden of specific conditions (Turner-Bowker *et al*. 2002). The SF-36v2 is scored according to clusters of questions and provides a physical health and mental health summary score.

### The intervention

In the study, the participants in the intervention group met with the researcher each week for 12 weeks for a 1 hour session. The sessions included education and discussion on the healthy lifestyle topic of the week (e.g. five food groups), and participants’ progress with the implementation of the healthy lifestyle components of the programme into their everyday life (Table 1: 12 weekly session topics). After the group education session, a 30-minute exercise activity, led by the researcher, was also undertaken. Groups met at the same time and place each week. During the development of the Passport 4 Life programme, it was important that consideration was given to some of the consequences of schizophrenia, particularly cognitive issues such as impaired memory and concentration difficulties, differing levels of literacy, and negative symptoms such as avolition, and a reduction in living and social skills (Robson & Gray 2007, Bardwell & Taylor 2009); all of which impact on ability to attend to learning situations. It was thus considered important to develop the education programme in a stepped, progressive way, that was easy to understand and delivered in a conversational tone. Visual reminders, such as healthy eating tips and pictorial representations, were also added throughout the programme to assist with learning (Park *et al*. 2011). The programme was conducted across a 12-week period to allow sufficient time for a weight change to be detected. For further detail on the intervention see Park *et al*. (2011).

**Tabel 1:** 12 weekly session topics

Week	Session details
1. Let’s get started	Welcome
	How to use the booklet
	Outline of 12 weeks
	How to use the pedometer
	Recording your success
2. Healthy eating choices	2 + 5 fruit & vegetables
	Healthy plate
3. Healthy snacks	How to choose healthy snacks
	What’s healthy?
	Making a healthy snack
	Suggestions
4. Recording and rewarding your success	Healthy celebrations – films, clothes
	Recording success
	Sharing your success
5. Exercise	Variety
	When to
	How to
6. Exercise choices	Making exercise fun
7. Healthy eating review	Reminders
	Review what works
	Review what doesn’t work
8. Feelings, exercise & eating–Part 1	What is the relationship?
9. Feelings, exercise & eating–Part 2	Who’s in control?
10. Evaluating your success	Comparing to others – positive & negative
11. How to keep motivated with the programme	Setting achievable goals
	Keep moving forward
12. Healthy celebrations	Review
	Reward
	Remind
	Evaluation
	Bon voyage!

### Procedure

Potential participants who met the study criteria were invited to attend the first session of each 12-week group series. After consenting to participate, all in attendance were invited to select an opaque envelope which provided the researcher with the random allocation of the participant. The result of the allocation was then explained to the participant. After a discussion about the conduct of the programme, participants were invited to complete the data collection tools with guidance from the research assistant as required. When all survey tools were completed, height, weight, and girth measurements were collected. Participants in the intervention group were provided with a copy of the education booklet, met every week for 12 weeks, and the measures were repeated at the completion of the programme. An overview of the programme has already been published (Park *et al*. 2011). Control group participants were provided with a copy of the education booklet and asked to return in 12 weeks for measurement outcome data collection. Control group participants were given contact details of the researcher if they required additional information. The study was conducted by a team of experienced mental health nurses who (with the consent of participants) were in contact with case managers as required throughout the study. The same nurse researcher led the intervention group activity for the duration of the project; this enabled a consistent approach.

### Ethical consideration

The study was approved by the relevant ethics committees and all participants received an explanation of the study before providing written consent. Participants in this study were deemed vulnerable due to their diagnosis of serious mental illness. Therefore, their consent to take part was only negotiated if they were deemed free of any symptoms of psychoses by an experienced mental health nurse at the time of entering the study.

### Data analysis

Numerical data were described using mean values and standard deviations (sd) or median values and inter-quartile ranges depending on the distribution. All characteristics assessed at baseline were compared between intervention and control participants to evaluate the effectiveness of randomization. Differences in primary outcome measures between baseline and 12 weeks follow-up (baseline values – week 12 values) were compared between intervention and control group using standard bivariate statistical tests, including Chi-squared tests, Fisher’s exact tests, unpaired *t*-tests, and non-parametric Mann–Whitney Wilcoxon *U*-tests. Statistical analysis was conducted using spss version 18 (SPSS Inc. Released 2009. PASW Statistics for Windows, Version 18.0. Chicago: SPSS Inc., Chicago, IL, USA).

## Results

Table 2 provides a detailed overview of the demographic information for all participants (*n* = 101) collected at the commencement of the study. There were slightly more male participants than females, with male participants making up 53·5% of the group.

**Tabel 2:** Participant demographic characteristics (*n* = 101)

Characteristic	Frequency	%
Gender		
Male	54	53·5
Female	47	46·5
Marital status		
Single	81	80·2
Married	3	3·0
Divorced	11	10·9
Separated	6	5·9
Ethnicity		
Caucasian Australian	72	71·3
Aboriginal or Torres Strait Islander	14	13·9
Other	15	14·9
First language spoken		
English	97	96·0
Other	4	4·0
Education level reached		
Completed high school	21	23·0
TAFE	11	10·0
Did not complete high school	54	49·1
University graduate	4	3·6
Current student	5	3·6
Current employment		
Paid work	5	4·9
Volunteer	12	11·8
Combination	3	2·9
Not working	79	78·2

The majority of participants (84·2%) self-reported a diagnosis of schizophrenia, with 15·8% of participants self-reporting bi-polar disorder, depression, or anxiety as their psychiatric diagnosis. Second generation medications taken by the participants included Olanzapine (36·3%), Risperidone (23·8%), Clozapine (18·8%), and Seroquel (14·9%) (Table 3). Attendance rates at the weekly sessions were high. The baseline results outlined in Table 4 indicate only small differences between intervention (*n* = 51) and control (*n* = 50) participants at the commencement of the study.

**Tabel 3:** Description of participants’ health characteristics (*n* = 101)

Characteristic	Frequency	%
Diagnosis		
Schizophrenia	85	84·2
Depression	7	6·9
Bi-Polar disorder	7	6·9
Anxiety	2	2·0
Current psychiatric medication		
Olanzapine	37	36·6
Clozapine	19	18·8
Risperidone	24	23·8
Seroquel	15	14·9
Amisulpride	2	2·0
Abilify	3	3·0
Avanza	1	1·0
Medical problems		
No	60	59·4
Yes	41	40·6
Surgical problems		
No	84	83·2
Yes	17	16·8
Case manager		
Mental health worker	63	62·4
None	36	35·6
Other	2	2·0

**Tabel 4:** Comparison of the participants (*n* = 101*) at baseline, stratified by intervention and control

Outcome measure	Control (*n* = 50)	Intervention (*n* = 51)	*P* value
Mean weight (sd) (kg)	97·7 (20·7)	97·2 (17·4)	0·891†
Mean girth (sd) (cm)	109·7 (14·1)	111·0 (11·8)	0·628†
Mean BMI (sd) (kg/m^2^)	34·0 (8·1)	33·3 (6·1)	0·601†
Self-reported medication compliance: active participation, *n* (%)	23 (50·0%)	23 (50·0%)	0·811‡
LUNSERS median of final score (IQR)	25 (13, 57)	28 (17, 47)	0·952¦
DAI median score (IQR)	4 (0, 8)	4 (0, 6)	0·954§
SF36 Mean physical score (sd)	55·6 (9·4)	57·4 (8·5)	0·303†
SF36 Mean mental health score(sd)	43·4 (5·8)	43·2 (6·3)	0·958†

* Not all participants answered all questions.

† unpaired *t*-test.

‡ Chi-squared test

§ Mann–Whitney Wilcoxon *U*-test.

Table 5 provides the BMI categories of all participants (*n* = 101) of the study at baseline. The majority of study participants (*n* = 89) had a BMI above the normal range, and the mean BMI of participants was 33·71 kg/m^2^ (sd 7·1) (Table 4). The current recommended healthy girth for men is <102 cm and for women <88 cm (IDF 2006). The mean girth of the study participants was 110·4 cm (sd 13·0).

**Tabel 5:** Body mass index (BMI) categories for all participants at baseline (*n* = 101)

BMI categories	Frequency
Normal range BMI: 18·50–24·9 kg/m^2^	12 (12·1%)
Overweight range: 25·00–29·9 kg/m^2^	25 (25·3%)
Moderately overweight: 30·00–34·9 kg/m^2^	21 (21·2%)
Severe obesity: 35·00–39·9 kg/m^2^	26 (26·3%)
Very severe obesity: >40·00 kg/m^2^	17 (17·2%)

The control group (*n* = 50) and intervention group (*n* = 51) were compared using differences in outcome measures between baseline and 12 weeks of follow-up (Table 6). The intervention group had a mean weight loss of 0·74 kg (sd 3·87, *P* = 0·167) in comparison to the control group who lost on average 0·17 kg (sd 3·36, *P* = 0·729). In both intervention and control groups seven participants each improved their medication compliance, while eight participants in the intervention and six participants in the control group had worse self-reported medication compliance at 12 weeks follow-up (*P* = 0·888).

**Tabel 6:** Results of outcome measures at 12 weeks of follow-up stratified by intervention (*n* = 51) and control (*n* = 50) participants*. Results of comparisons of differences between baseline and follow-up measurements

Outcome measure	At baseline	At 12 weeks follow-up	Difference between baseline and follow-up	*P* value
Mean weight (sd) [kg]				
Control (*n* = 50)	97·7 (20·7)	97·5 (20·7)	0·17 (3·36)	0·420†
Intervention (*n* = 51)	97·2 (17·4)	96·4 (17·2)	0·74 (3·78)	
Mean girth (sd) [cm]				
Control (*n* = 50)	109·7 (14·1)	109·6 (14·6)	0·15 (3·22)	0.217†
Intervention (*n* = 51)	111·0 (11·8)	109·8 (11·5)	1·23 (5·24)	
Mean BMI (sd) [kg/m^2^]				
Control (*n* = 50)	34·0 (8·1)	33·9 (8·1)	0·06 (1·17)	0·435†
Intervention (*n* = 51)	33·3 (6·1)	33·0 (6·0)	0·25 (1·34)	
Self-reported medication compliance: active participation, *n* (%)				
Control (*n* = 50)	23 (50·0%)	24 (52·2%)	Improved: 7 (16·7% of 42)	0·888‡
Intervention (*n* = 51)	23 (50·0%)	22 (46·8%)	Improved: 7 (15·6% of 45)	
LUNSERS median final score (IQR)				
Control (*n* = 50)	25 (13, 57)	15 (7·5, 26·5)	6·0 (−5·25, 24·5)	0·066§
Intervention (*n* = 51)	28 (17, 47)	25 (13, 37)	0·0 (−13·0, 14·0)	
DAI median score (IQR)				
Control (*n* = 50)	4 (0, 8)	6 (2·5, 8)	0·0 (−2·0, 0·0)	0·739§
Intervention (*n* = 51)	4 (0, 6)	6 (2, 8)	0·0 (−2·0, 2·0)	
SF36 Mean physical score (sd)				
Control (*n* = 50)	55·6 (9·4)	57·5 (8·8)	−1·96 (9·44)	0·397†
Intervention (*n* = 51)	57·4 (8·5)	57·9 (8·0)	−0·34 (9·08)	
SF36 Mean mental health score (sd)				
Control (*n* = 50)	43·4(5·8)	44·3 (5·7)	−0·64 (6·99)	0·797†
Intervention (*n* = 51)	43·2(6·3)	44·0 (6·2)	−1·02 (6·90)	

* Not all participants answered all questions.

† unpaired *t*-test.

‡ Chi-squared test.

§ Mann–Whitney Wilcoxon *U*-test.

## Discussion

The results of the study were not statistically significant, however, small changes in the predicted direction did occur for the intervention group. Individual or group lifestyle approaches have been reported as achieving modest weight loss and are recommended at the commencement of treatment with second generation antipsychotic medications (Álvarez-Jiménez *et al*. 2008). Multi-modal approaches, which include a combination of education, healthy lifestyle, behavioural, and exercise interventions, are more likely to be effective in reducing weight in the longer term than any of the approaches on their own (Poulin *et al*. 2007). The intervention used in the study included a healthy lifestyle, exercise, and motivational interviewing approach. The failure to find a significant result can be attributed to a number of factors. The participants in the study had all been taking Second generation antipsychotic medication for some time. It is possible that their weight had reached a plateau, which may have made weight-loss less likely. Furthermore, previous interventions that combined a variety of weight-loss techniques, such as exercise, diet, and education, that were conducted over a longer period of time, 16 weeks (Weber & Wyne 2006), 18 weeks (Richardson *et al*. 2005), 6 months (Chen *et al*. 2009; 18 months (Poulin *et al*. 2007), were more likely to report a significant result. Perhaps the study reported here would have a significant result if the intervention had been conducted over a longer period of time. It is also possible that change in the control group had an impact of the study outcome. Both the intervention and control group were provided with a specially designed educational booklet that provided the participant with healthy eating hints, menu planning tips, and weekly menu planners (Park *et al*. 2011). This information may have influenced the control group behaviour and resulted in weight loss in that group overall. Content and comprehension of education programmes is a critical factor for success (Park *et al*., 2011). It is possible that the educational programme devised and delivered did not sufficiently inform the participants about how to make the required changes to lead to weight reduction. Furthermore, the once weekly exercise sessions that followed the education sessions may have been inadequate for weight change in participants who did not undertake any further exercise during the week.

Motivational interviewing was added to the intervention to complement the approach. Acknowledging that the most successful weight-loss interventions include a combination of approaches rather than any individual approach on its own (Poulin *et al*. 2007), the intervention was designed to be inclusive of education, exercise, lifestyle change, and diet. In addition, the intervention was designed using the principles of motivational interviewing. Given that the result of the study was not statistically significant, it is possible that this technique made little difference to the overall outcome or that the use of the motivational technique needs to be offered more than once each week for behaviour change to occur.

In addition to the weight loss results, the participants at baseline and 12 weeks scored higher than the norm for age with regard to physical health on the SF36. This result indicates that the participants initially considered themselves to be relatively ‘physically healthy’. However, the physical measurements of the participants at baseline and 12 weeks indicate that the majority had a girth higher than the recommended healthy range and a BMI score above the normal recommended range. Therefore, the participants of this study were already overweight; this is similar to findings from previous studies such as Smith *et al*. (2007) who reported a study of 966 people with serious mental illness and found overall the BMI of participants was higher with a mean of 46 kg/m^2^.

## Study limitations

The study was not blinded as both the researcher who delivered the intervention and the participants knew whether they were in the intervention or control group. Failure to blind the researcher and participants in intervention studies is common (Schneider *et al*. 2007), and in this study it was only possible to blind the research from the data collected by using a research assistant to collect measurements.

Location of the study groups was also a limitation of the study. Some of the intervention groups were conducted close to parks and areas where recreation activities were easy to find while others were in less than ideal locations. For example, one group was conducted at a setting where there is a gym located on-site, however, it is not air-conditioned and was not always available for use by the participants.

The ability of the participants to comprehend the information presented in the educational programme was not formally tested. This may have had an impact on the outcome of the study if the participants were unable to understand how to improve their eating and dietary habits.

The participants were recruited via advertisement at local non-government organizations and through the local health service. It is possible the sample may have been skewed towards a particular representation of people with serious mental illness as a result and perhaps other recruitment procedures would lead to different outcomes.

**What is already known about this topic**

- Weight gain has reached epidemic proportions in the general population but people with mental illness are at greater risk of weight gain, especially those taking second generation antipsychoticmedications.
- People taking second generation antipsychotic medications gain weight at a predictable rate and lifestyle interventions designed to help manage weight gain are more effective than pharmacological interventions or standard care groups.

**What this paper adds**

- A lifestyle, education and weight loss intervention undertaken in combination with motivational interviewing conducted over 12 weeks did not result in significant weight loss.
- A similar intervention should be undertaken over an extended period of time as the results indicated weight change in the predicted direction even though not significant.

**Implications for practice and/or policy**

- Mental health nurses must to take the lead in assessment and management of physical health concerns for people with serious mental illness.
- Individual components of the program could be implemented in different settings, such as the education component could be delivered as a group program in inpatient and community settings.
- Mental health nurses need to increase their knowledge of assessment and management of medication side effects, such as weight gain for people with serious mental illness.
- Future research should involve qualitative studies to gain a deeper understanding of the experience of medication induced weight gain.

## Conclusion

The study reported was developed due to the increasing body of evidence in the literature linking weight gain for people with serious mental illness to the prescription of second generation antipsychotics. The results of the study demonstrated that healthy lifestyle changes could have a positive effect on weight. The observations of the researcher found a willingness of the participants to be engaged in healthy lifestyle changes. It is imperative that mental health nurses act on the mounting body of evidence and start to include healthy lifestyle education at the commencement of antipsychotic medications rather than wait until weight gain has become problematic. Additional research is needed to understand how individual components of weight change need to be incorporated to bring about change and how best mental health consumers can be assisted to manage the weight gain associated with second generation antipsychotic medications.
